# Does Socioeconomic Status Affect Patients’ Ease of Use of a Touch-Screen (iPad) Patient Survey?

**DOI:** 10.2196/ijmr.2314

**Published:** 2013-01-11

**Authors:** Saman Zarghom, David Di Fonzo, Fok-Han Leung

**Affiliations:** ^1^University of TorontoFaculty of MedicineToronto, ONCanada; ^2^University of TorontoFaculty of Kinesiology and Physical EducationToronto, ONCanada; ^3^University of TorontoDepartment of Family and Community MedicineToronto, ONCanada

**Keywords:** socioeconomic factors, age factors, medical informatics, computer-user interface

## Abstract

Socioeconomic disparities influence the usage rate of advanced communication technologies in Canada. It is important to assess all patient interactions with computers and electronic devices based on these socioeconomic differences. This project studied the ease of use of a touch-screen interface program for collecting patient feedback. The interface collected feedback on physicians’ communication skills, an important health concern that has been garnering more and more attention. A concurrent paper survey was used to gather information on the socioeconomic status and the usability of the touchscreen device. As expected, patients who were older, had lower annual household income, and had lower educational attainment were associated with more difficulty using the devices. Surprisingly, 94% of all users (representing a wide range of socioeconomic status backgrounds) rated the device as easy to use.

## Introduction

Education and literacy are important determinants of health. Unemployment, poverty, and poor health are more common amongst Canadians with low literacy rates [1]. Income and educational level also influence the usage of the new communication technology. The use of the Internet by Canadians is influenced by income, education, and age [1]. This research project studied the usability of a new touch interface program for collecting patient feedback. The feedback focused on physician communication skills [2-5] as part of a larger initiative to target staff and resident education. Previous studies have demonstrated patient opinion as a reliable proxy for the strength of physician communication skills [2,3]. This current study looks at the feasibility of using touch screen interfaces for patients, examining touch screen use, and socioeconomic markers. We wanted to ensure that, prior to implementing touch screen interfaces more widely, that its use would be equitable. 

## Methods

The target population of this study was all of the English-speaking patients over 18 years of age who received medical care from the 80 Bond Street family clinic in Toronto, Canada, between January 1 and March 1, 2011. The local research ethics board approved the study. To assess the usability of the touch screen interface and to collect the socioeconomic status data of the sample population, a paper-based survey was developed and used in conjunction with the touch screen. Following routine registration, patients were approached in the waiting room with a touch screen device. A convenience sample was collected; every patient in the waiting room was approached. After consenting, patients used the touch screen device to answer questions on physician communication skills. Patients were then provided a paper survey inquiring about device usability. Responses on the paper surveys were analyzed using Pearson’s chi-square test without Yates correction. Statistical significance was set at *P*<.05.

## Results

490 patients were approached for the study with a 72% response rate (N=353) representing a broad range of socioeconomic statuses, while 130 declined and 7 participants were excluded (non-responders, Table 1). All results were patient self-reported.

**Table 1:** Demographic information of electronic feedback users.^a^

Category		n (%)
**Age**		
	<50	224/352 (63.6)
	≥50	128/352 (36.4)
**Gender**		
	Male	168/348 (48.3)
	Female	180/348 (51.7)
**Income**		
	<50k	168/329 (51.1)
	≥50k	161/329 (48.9)
**Education**		
	No university degree	172/348 (49.4)
	University degree	176/348 (50.6)

^a^note variation in sum of numbers due to non-responders

### Ease of Use

Ease of use was a patient self-reported measure. Older age (≥50 years), lower income (<$50,000), and lower educational status (*P*=.03) were associated with statistically significant difficulty using the touch screen device (Table 2). Conversely, younger age (<50 years) (*P<*.001), higher income (≥$50,000) (*P<*.001), and higher educational status (university/college degree completed) (*P*=.03) were associated with significant ease of use for the touch screen device (Table 2).

**Table 2:** Ease of use rating by patients.^a^

Category		Very easy/easy n (%)	Neutral/difficult n (%)	*P* value
**Age**				
	<50	214/221(96.8)	7/221(3.2)	<.001
	≥50	104/127(81.9)	23/127 (18.1)	<.001
**Income**				
	<50K	114/138(82.6)	24/138 (17.4)	<.001
	≥50K	188/192(97.9)	4/192 (2.1)	<.001
**Education**				
	No university degree completed	154/175(88.0)	21/175 (12.0)	.03
	University degree completed	160/169(94.7)	9/169 (5.3)	.03

^a^note variation in sum of numbers due to non-responders

### Likelihood of Reuse

Older age (≥50 years), and lower educational status (no university/college degree completed) were associated with significant likelihood of not reusing the touch screen device (Table 3). Conversely, younger age (<50 years), and higher educational status (university degree) were associated with significant likelihood of reusing the touch screen device (Table 3).

**Table 3:** Likelihood of reuse rating by patients.^a^

Category		Very likely/likely n (%)	Neutral/unlikely n (%)	*P* value
**Age**				
	<50	193/221(87.3)	28/221(12.7)	<.001
	≥50	90/125(72.0)	35/125(28.0)	<.001
**Income**				
	<50K	131/165(79.4)	34/165 (20.6)	.32
	≥50K	133/159(83.6)	26/159(16.4)	.32
**Education**				
	No university degree completed	133/173(76.9)	40/173 (23.1)	.02
	University degree completed	146/169(86.4)	23/169 (13.6)	.02

^a^note variation in sum of numbers due to non-responders

### Likelihood to Recommend

Older age (≥50 years) was associated with significant likelihood of not recommending use of the touch screen device (Table 4). Conversely, younger age (<50 years) was associated with significant likelihood of recommending use of the touch screen device (Table 4).

**Table 4:** Likelihood to recommend by patients.^a^

Category		Yes n (%)	No n (%)	*P* value
**Age**				
	<50	213/217 (98.2)	4/217 (1.8)	.01
	≥50	114/123 (92.7)	9/123 (7.3)	.01
**Income**				
	<50K	152/162 (93.8)	10/162 (6.2)	.06
	≥50K	153/156 (98.1)	3/156 (1.9)	.06
**Education**				
	No university degree completed	164/170 (96.5)	6/170 (3.5)	.75
	University degree completed	160/167 (95.8)	7/167 (4.2)	.75

^a^note variation in sum of numbers due to non-responders

## Discussion

As one might intuit, our results show that older age, lower income, and lower educational attainment were factors associated with significant difficulty using the touch screen device when compared with those that are younger, with greater income, and with greater educational attainment. The surveyors observed that some of the older users, particularly those with motor difficulties (eg, tremor), seemed to struggle to adapt to the sensitivity and responsiveness of the touch screen. Older age was also associated with lower chances of using the program in future visits and recommending the program to others. These findings point to the importance of maintaining routes for patient feedback other than touch screens—while touch screens present significant efficiencies in the collection and collation of patient feedback data, patient equity must also be considered.

While there was a statistically significant difference in the patient ratings when considering age, income, and education, it should be noted that there was an overall high rating for ease of use. The participation/response rate was also very high. These findings very strongly suggest that touch screen technology can play an important role in acquiring successful patient surveys. While there is scant research on the use of touch screens in clinical waiting rooms, the existing literature on human computer interactions and interfaces supports the increased use of touch screens [6]. This study was performed in an inner city clinic, and given the overall high ratings (owing to the statistically significant findings), the results indicate that using touch screen technology for patient feedback is feasible. Furthermore, given the globally high participation rate and positive results, touch screen technologies might also play a role in encouraging health consumer equity.

In using a convenience sample, some selection bias could have been introduced. The study only considered one clinical setting. The sample size was also limited. Further study is warranted. However, this study answered an important feasibility question. Touch screen interfaces can be easy to use, and can represent an accessible way for patients to provide feedback. This has implications for all clinics interested or engaged in quality initiatives to enhance patient satisfaction with their physicians.
